# Interactions between vertical migration and local oceanography drive microplastic exposure for Antarctic krill

**DOI:** 10.1038/s41598-026-50531-0

**Published:** 2026-05-16

**Authors:** Katherine L. Gallagher, Clara Manno

**Affiliations:** 1 School of Marine and Atmospheric Sciences, Stony Brook University, Stony Brook, NY 11794 USA; 2 British Antarctic Survey, Cambridge, UK; 3 Present Address: IBSS Corporation in Support of NOAA Fisheries, Silver Spring, MD 20910 USA

**Keywords:** Climate sciences, Ecology, Ecology, Environmental sciences, Ocean sciences

## Abstract

Euphausiids play important roles in ecosystems worldwide, transferring energy from primary producers to secondary consumers. Antarctic krill are the most abundant euphausiid on Earth and play important roles as critically important species in Southern Ocean ecosystems. Microplastics have been found throughout the Southern Ocean ecosystem, including krill. Here, we examine where krill and microplastics interact throughout the coastal ocean of the West Antarctic Peninsula. Using a physical ocean model to simulate the transport of vertically migrating krill and drifting microplastics, we identified several hotspots around the Antarctic Peninsula where both are consistently found in higher than average abundances. The extent of the overlap was modulated by both currents in the near-surface (< 50 m) and deep (> 50 m) ocean, and the vertical migration behaviors of Antarctic krill. Therefore, in order to understand the impacts of microplastics, we need to evaluate overlap in the context of euphausiid behavior and oceanographic conditions.

**Supplementary Information:**

The online version contains supplementary material available at 10.1038/s41598-026-50531-0.

## Introduction

Euphausiids (henceforth krill) are small, planktonic crustaceans found in all the world’s oceans^1^. Antarctic krill (*Euphausiia superba*) occupy the Southern Ocean from the deep ocean to the continental shelf^2,3^. They are the most abundant euphausiid on Earth, with an abundance of 300–500 million tons^4^. They are critical species for local food webs; several species of seabirds, whales, and seals primarily consume Antarctic krill during the austral summer when biological activity is greatest^5,6^.

Antarctic krill also provide several ecosystem services. These include serving as critical facilitators of biogeochemical cycles and carbon sequestration^7–9^. By consuming phytoplankton and vertically migrating to deep waters, Antarctic krill contribute to carbon export through the production of fast-sinking faecal pellets, continuous shedding of exuviae and sinking carcasses, all of which transport organic carbon from surface waters to the deep ocean. The remineralization of these pellets by krill, bacteria, or other zooplankton can help facilitate carbon, nitrogen, phosphorus, and iron cycling^7–9^. Krill also support fishing industries worldwide and are used for supplements, animal feeds or bait, or human consumption^5,10^.

Despite their distance from human civilizations, Antarctic krill, like their counterparts in other oceans, still face anthropogenic threats and stressors. Warming climates have reduced the extent of sea ice. Both larval and juvenile Antarctic krill depend in part on sea-ice habitats during winter, which provide critical food resources, shelter, and support for growth and survival^5,10–15^. As a result, lower concentrations of sea ice reduce krill spawning success^15,16^. Changing climates have also altered the distributions and size of krill prey, with conditions occasionally favoring smaller phytoplankton that krill cannot consume^17^, and increased bloom frequency in salps, which can compete with krill^16^.

Microplastics (1 -1000 m^18^) have been found across the Antarctic ecosystem, from snow and ice to penguin guano, and Antarctic krill are no exception^19–29^. Acute toxicity of microplastic consumption in Antarctic krill is limited, but the long-term effects are still poorly understood^30^. Antarctic krill can also break down microplastics into nanoplastics (< 1 m)^21,28^, which can impact development and molting behaviors, as well as larval development and lipid reserves^31–33^. Furthermore, there is growing concern that microplastics in Antarctic krill fecal pellets may impact carbon sequestration and biogeochemical cycles by reducing sinking rates and altering remineralization rates^7,34,35^.

Microplastic concentrations within the Southern Ocean are believed to be significantly lower than other ocean basins^36^. In the Atlantic sector, where sampling efforts are greatest, surface concentrations of microplastics are generally higher closer to shore^37,38^. Subsurface microplastics distributions are believed to exponentially decay with depth^31^. Paint fragments make up a majority of observed pollutants, suggesting that fishing, tourism, research vessels, and long-range transport are significant sources of pollution and microplastics to the region^37–39^. Research stations may also play a large role on local scales, due to microplastic pollution from personal care products and laundry facilities^39,40^. Other than paint fragments, the most common microplastics observed in the region are fibers and fragments, most frequently made up of polyurethane, polyethylene, and polyester polymers^31,37–39^. However, a majority of sampling campaigns only sample the surface ocean, methods are not standardized across sampling efforts, and efforts are not uniformly distributed across space and time^39,41^. Therefore, it is challenging to understand the true distribution of microplastics in the region, and, as a result, understand where Antarctic krill and microplastics may be interacting to build effective mediation and monitoring efforts.

Antarctic krill, like many other polar species, are adapted for relatively stable environments. With stressors compounding in these environments, there is a concern that a species’ ability to adapt will decrease^32,34,39^. Therefore, it is important that we understand the spatial distribution of potential stressors. A recent study by Hunter and colleagues^41^ found that krill were at the greatest risk for interacting with microplastics along the Antarctic Peninsula, and suggested that the presence of climate stressors (temperature change and ocean acidification) in the region may exacerbate the impacts of microplastics on the ecosystem. While this work highlighted potential overlaps on a circumpolar scale, they did not consider the impacts of krill or microplastic depth distributions^41^.

**Fig. 1:**
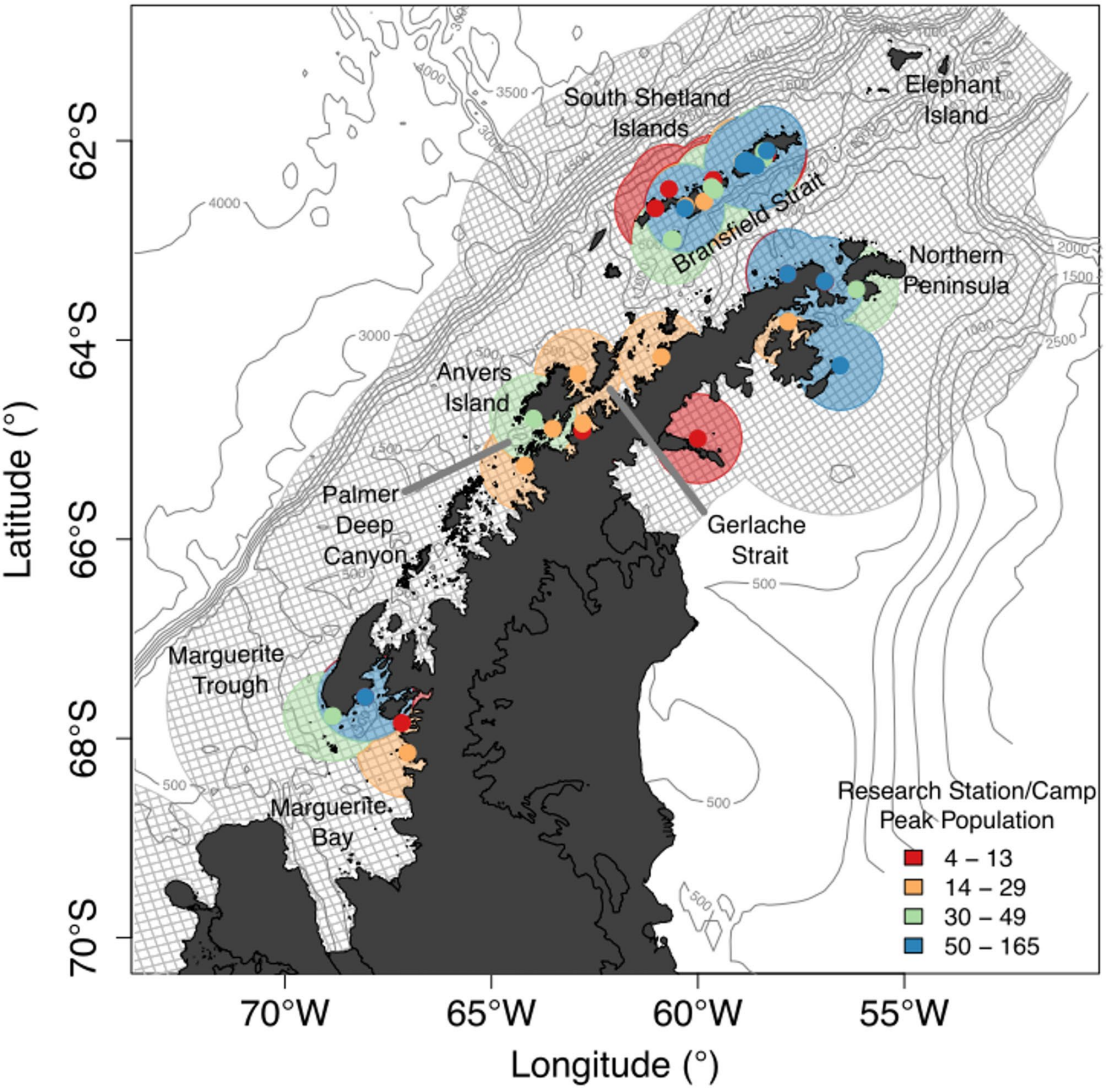
A map of the West Antarctic Peninsula illustrating the study area where simulated krill and microplastics were released, model bathymetry, the 10 × 10 km grid used to count simulated krill and microplastics, the locations of research stations and camps (points), and the 50 km buffer regions around research stations and camps. Points and polygons representing stations and camps and the areas around them are colored based on the number of occupants, divided into 25% quantiles. Microplastics were released in these areas at different densities based on the number of occupants, with fewer microplastics released around smaller research stations.

Antarctic krill however, are found throughout the water column and, like many euphausiid and zooplankton species worldwide, exhibit diel vertical migrations^42–48^. This behavior, where krill migrate to the surface ocean at night to feed and retreat to the deep ocean during the day to avoid visual predators^47,48^, can significantly modulate krill distributions, especially in systems where ocean currents promote retention^49,50^. Therefore, the vertical location of both Antarctic krill and microplastic pollution, may significantly alter the overlap between krill and microplastics. Using simulated krill and microplastics advected in a regional physical ocean model, we quantify how krill vertical migration behaviors alter the overlap in krill and microplastic accumulation hotspots along the West Antarctic Peninsula. We show that krill and microplastic hotspots persistently overlap in the Bransfield Strait, the northern Peninsula, and Marguerite Bay. This overlap differed depending on the vertical migration behaviors of the simulated krill, highlighting how different current systems in the upper (< 50 m) and lower (> 50 m) ocean will play a role in how krill interact with these pollutants. These areas of high overlap represent not only areas of high krill predator activity, but also represent areas where additional anthropogenic activities through scientific research and fishing impose additional stressors in an already stressed, fragile ecosystem where anthropogenic activities are forecasted to increase in the near future (Fig. 1).

## Results

### Krill and microplastic hotspots

**Fig. 2:**
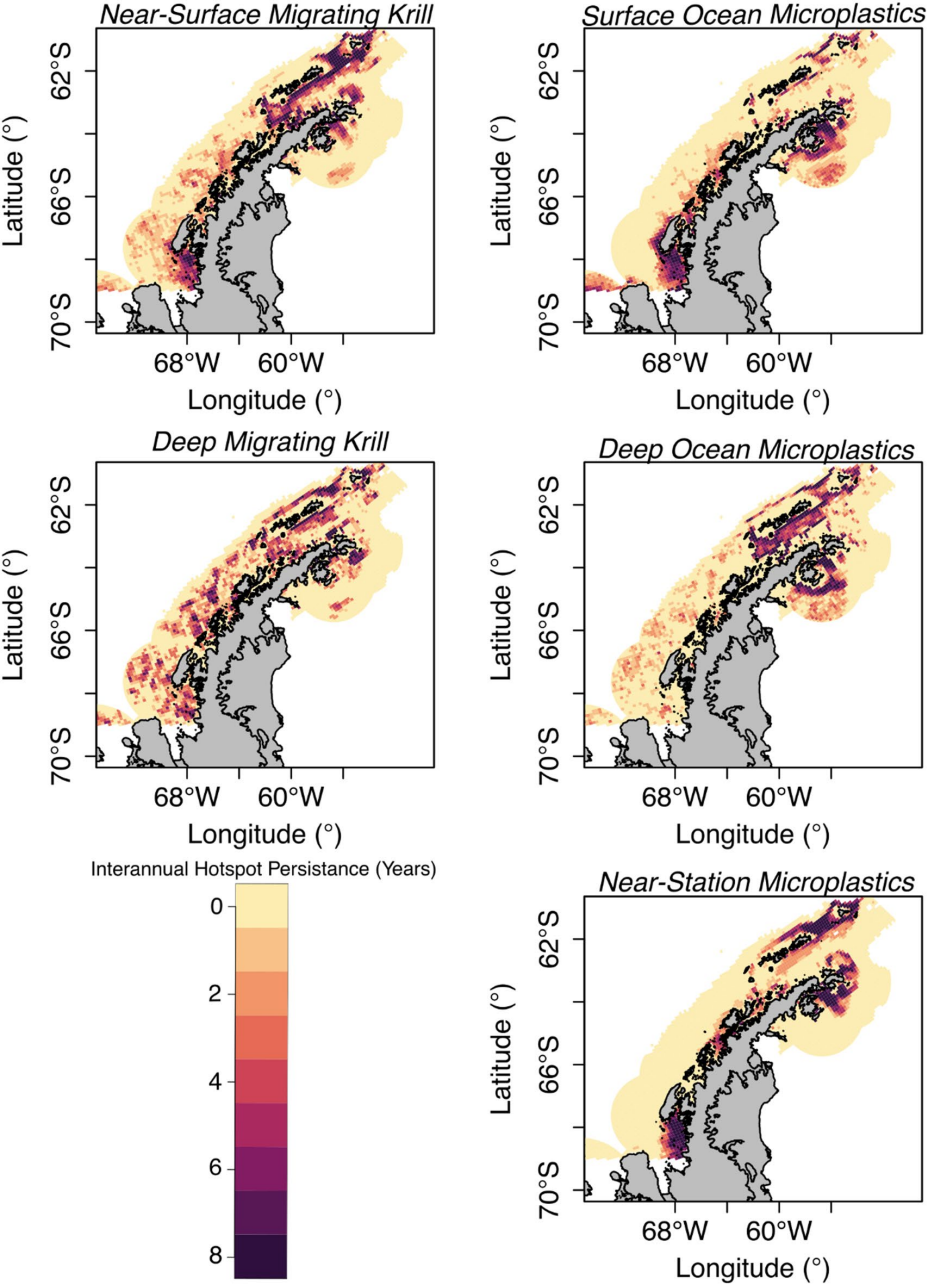
Interannual hotspot persistence in years for krill (left column) and microplastics (right column) where darker colors indicate that the grid cell was a hotspot in more of the simulated years.

Krill hotspots were the most common along the South Shetland Islands in and around the Bransfield Current System, the northern tip of the Peninsula. Krill hotspots were also common in Marguerite Bay and along the continental shelf southwest of Anvers Island (Figs. 2, S1-2).

Microplastic hotspots were persistent within Marguerite Bay, around the northern tip of the Peninsula, as well as some portions of the Bransfield Strait around the South Shetland and Elephant Islands (Figs. 2, S3-5). When microplastics were released in deeper waters (> 50 m), hotspots were more common in the Bransfield Strait and the northern tip of the Peninsula (Figs. 2, S4). When microplastics were released around existing research stations and camps, hotspots were consistently found around the South Shetland Islands, within Marguerite Bay, as well as around the northern tip of the Peninsula and in the Gerlache Strait (Figs. 2, S5).

**Fig. 3:**
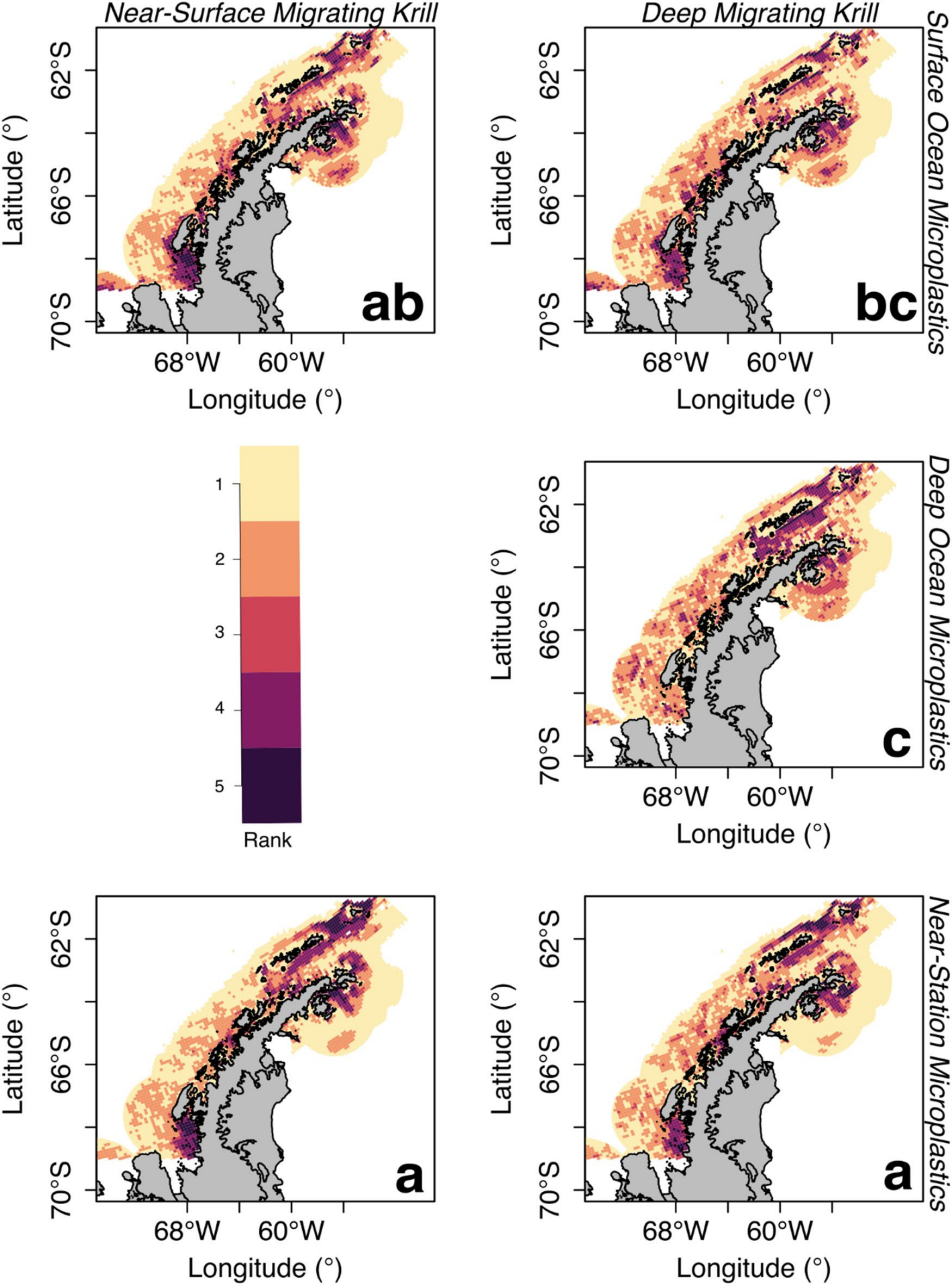
Overlap of krill migrating within the surface ocean (left column) and to the deep ocean (right column) and microplastics released in the surface ocean (top row), deep ocean (middle row), and near research stations and camps (bottom row). Krill migrating within the surface ocean and deep microplastics were not compared since they would not interact. Letters indicate differences in ranks as determined by Kruskal-Wallis and pairwise Wilcoxon tests (*p* < 0.05), such that panels containing different letters are significantly different from each other.

### Distributions of krill and microplastic hotspots

Overlap in krill and microplastic accumulation were greatest in areas such as the South Shetland Islands, the northern Peninsula, and Marguerite Bay (Figs. 3, S6-10). Overlaps were similar when microplastics were only released in the surface ocean, whereas overlap was higher in the Bransfield Strait and northern Peninsula when microplastics were released in deeper waters (Fig. 3). Krill and microplastics overlaps were greatest when microplastics were released from around research stations and camps in both the Bransfield Strait and Marguerite Bay (Fig. 3).

The amount of overlap between krill and microplastics varied significantly across microplastics sources and krill migration behaviors (Kruskal-Wallis test, chi-squared = 46, df = 4, p < < 0.01). While overlap did not differ across microplastic depths when krill migrated within the surface ocean, overlap did differ across microplastics depths when krill migrated to depth (Fig. 3, p < < 0.01). Overlap between deep migrating krill and deep microplastics and microplastics released near research stations differed (Fig. 3, p < < 0.01). The overlap between deep migrating krill and deep microplastics also differed from overlaps with near-surface migrating krill and microplastics released near the surface and near stations (Fig. 3).

## Discussion

We identified several areas along the West Antarctic Peninsula where the overlap of microplastics and krill was high: the Bransfield Strait between the South Shetland Islands and the Peninsula, the northern tip of the Peninsula, south of Anvers Island, and Marguerite Bay. We also found that the vertical migration behavior of krill, and the depth of microplastics made a significant difference in the location and intensity of overlap. The overlap between krill and microplastics in the Bransfield Strait, including along the south coast of the South Shetland Islands, and between the South Shetland and Elephant Islands, persisted across krill vertical migration behaviors, but had the greatest spatial extent when krill migrated deep (below 50 m) and microplastics were released below 50 m. Around the northern tip of the Peninsula, the overlaps were greatest when microplastics were released in near-surface waters and krill migrated within the surface ocean, and around research stations, regardless of krill migration behavior. South of Anvers Island, the greatest overlaps were observed when krill migrated deep, and microplastics were released around research stations and camps. In Marguerite Bay, the spatial overlap between krill and microplastics was greatest when krill migrated and microplastics were released within the surface ocean.

Current systems in many of these regions are highly retentive, especially at depth with previous studies illustrating that simulated krill with vertical migration behaviors transported into the region can be retained in these for 10–20 days^51^. Flow below the mixed layer across the Peninsula in the region is barotropic (bathymetry-following) so the presence of steep bathymetry in Bransfield Strait and Palmer Deep Canyon result in the formation of persistent current features at depth that promote retention^51–56^. These persistent current features resulted in more overlap between krill and microplastics, especially when krill migrated out of the surface mixed layer and into the more retentive waters below.

Marguerite Bay, in comparison, is shallower and lacks the steep bathymetry of Palmer Deep Canyon or the Bransfield Strait. In the absence of these features in Marguerite Bay, there are likely fewer consistent current features to retain krill and microplastics below the mixed layer. Marguerite Trough is a relatively deep submarine canyon that cuts across the continental shelf from the shelf break to the Bay, and has been shown to provide a consistent source of krill to the region^51,57^. The Trough, therefore, may also provide a constant source of microplastic pollutants. Once krill and microplastics reach Marguerite Bay, however, it is likely that tidal processes and prevalent winds drive near surface current patterns that promote the observed retention of near-surface krill and microplastics. The Antarctic Coastal Current, which moves along the coast in this region^57,58^ may also play a role in the retention of krill and microplastics within the mixed layer.

The high overlap between krill and microplastics observed in the northern Peninsula, however, is not associated with any bathymetric features that may help promote retention. Previous studies have illustrated that krill can be retained in this region by the Coastal Current moving out of the Weddell Sea and a southern return flow between the tip of the Peninsula and D’Urville and Joinville Islands^51^. While the persistence of this southward flow between the Peninsula and the islands is unclear^53,59,60^, it is likely that this same baroclinic (buoyancy-driven) current system is playing a role in the overlap of krill and microplastics observed in this region. Similar to Marguerite Bay, surface wind-driven and tidal currents may also play a role, especially in the mixed layer where overlaps between krill and microplastics were greatest.

Vertical migration behavior was only performed to set depths throughout our simulations and was consistent throughout the year. While krill have been observed migrating to these depths along the Peninsula previously^49^, this behavior is also highly variable in Antarctic krill throughout the Southern Ocean^42,43,45,46,61,62^. In addition, diel vertical migration in krill has been shown to vary within a single season in correlation to day length, and not all krill perform these migrations every day^45,46,49^. Therefore, our simulations are a simplified version of this behavior. Future work should include (1) improving our understanding of krill vertical migration behaviors and their variability through long term acoustic mooring deployments throughout the region and (2) improving the krill model to make this behavior more realistic by adding realistic krill horizontal and vertical movement^63^.

It is also important to note that the water column is not the only place where krill can potentially interact with microplastics. Microplastics have been found in seafloor sediments worldwide^64^. Krill have been observed interacting with the seafloor along the Antarctic Peninsula^65^, but why krill may migrate to and interact with seafloor sediments, as well as the concentration of microplastics within these sediments along the Peninsula is poorly understood. Therefore, it is unclear whether or not seafloor sediments could serve as a significant source of microplastics to krill. Krill do, however, feed on the bottom of sea ice in the austral winter^66^ and winter sea ice extent is directly linked to krill recruitment success^5,67,68^. Microplastics have been found within sea ice across the Southern Ocean^27^ and could therefore be a significant source of microplastics to krill that we did not consider here.

One additional mechanism that plays a role in the distribution of microplastics and therefore may impact where these pollutants and krill overlap are the properties of the microplastics. Here, we treated the simulated microplastics as passive drifters and did not define any sinking or buoyancy properties that would be associated with different plastic types or shapes. Previous studies have classified the composition and shape of microplastics around the Peninsula^31,37,69,70^ and the buoyancy properties of different plastic types, and the impacts of composition on microplastics transport are known^71^. However, comprehensive, systematic studies that both classify the distributions and compositions of microplastics are rare in the Southern Ocean^39,41^, so the true distributions of different types of microplastics are poorly understood. Future work would include improving our understanding of the distribution of different types of microplastics, so that future simulations could be improved. For example, polyethylene, polypropylene, and polyester fibers were the most common microplastics found in Antarctic krill^19,29^. Polyethylene and polypropylene are slightly less dense than sea water, so adding a floating behavior or allowing particles to remain at a neutral buoyancy would improve future modeling efforts^71^. In contrast, polyester is denser than seawater, so adding a sinking behavior to simulated microplastics will improve future modeling efforts^72^.

Antarctic krill are threatened by a variety of anthropogenic stressors, such as a growing krill fishing industry. The areas of high overlap between the South Shetland Islands and the Peninsula also spatially coincide with areas of high activity within the Antarctic krill fishery^73,74^. While these waters are seasonally protected by a voluntary closure around the coasts near penguin colonies^74^, this also represents an area where microplastics can be consumed by Antarctic krill, which are then caught by the fishery for human consumption or by predators^20,24^.

In addition to fishing, human activities in the Southern Ocean, especially along the Peninsula, are increasing through scientific research^75^ and tourism^76^ and these activities potentially serve as important local sources of plastic pollution^40,69,70,77–79^. Microplastics released around research stations and camps overlapped with krill hotspots in Marguerite Bay, south of Anvers Island, around the northern Peninsula, and around the South Shetland Islands. While the intensity of the overlap varied slightly with krill migration behaviors, there were no significant differences in the overlap patterns observed. All of these areas contain several, high-capacity (peak populations ranging between 30 and 165 individuals, which represents the upper 50% of station peak populations within the region) research stations that could be sources for these pollutants^80^. Many of these research stations are open year-round (15 out of 22 stations with populations in the upper 50% of all research stations and camps)^80^. While populations in the region generally peak in the austral summer, which overlaps with our simulations, the year-round operation of these high-capacity research stations illustrates that these areas may also serve as microplastic hotspots year-round. However, it is important to note that our simulations only illustrate where microplastics are likely to accumulate, and are not reflective of true abundance. They instead highlight the potential for local source pollution from these research stations and camps. While we did not directly examine potential pollution from tourism activities here, previous studies have shown that macroplastic (> 1000 m^18^) pollution near the surface from tourism activities have the potential to accumulate broadly across the Peninsula^77,81^.

Euphausiids worldwide face similar anthropogenic stressors from multiple sources, including microplastics, and the accumulation of multiple anthropogenic stressors will likely impact their overall fitness^34,82^. We have illustrated that the interactions between vertical migration and local oceanography can change the extent to which krill are exposed to microplastic pollution. Therefore, in order to accurately estimate and mitigate exposure risk, we must consider both vertical migration behaviors and local oceanographic processes holistically. Unfortunately, vertical habitat use and migrations in euphausiids is highly variable, often changing on seasonal to daily timescales. Furthermore, the vertical distributions and transport mechanism of microplastics are poorly understood, with few systematic studies worldwide. Improving our understanding of vertical habitat use in euphausiids and the vertical distributions of microplastics, and pairing this knowledge with regional ocean models, will help understand how these critical components of global ecosystems are exposed to microplastic pollutants, allowing managers to build effective mitigation actions and marine protected areas to preserve these ecosystems in the face of increasing anthropogenic stressors.

## Methods

### Model parameters and particle release schemes

Krill and microplastics were simulated within the Regional Ocean Modeling System (ROMS) for the West Antarctic Peninsula^83,84^. This iteration of the model has a 1.5 km horizontal resolution and 24 vertical terrain-following layers^84^. Tides are included in the model and include nodal corrections as needed^85^. The model’s atmospheric forcing is from the Antarctic Mesoscale Prediction System (AMPS)^86^, and interactions between ice shelves, sea ice, and the underlying waters are included^87,88^. We simulated eight austral summers (November – March) between 2006 and 2012 and 2018–2020 following previously published work^89^.

Diel vertical migration (DVM) behaviors were added to passive particles within ROMS to simulate krill following previous studies^49–51,89^. We simulated DVM using local sun angle, where simulated krill swam upwards to a set depth when sun angles were negative during the night, and swam downwards to a set depth when sun angles were positive during the day. Vertical swimming speeds were set to 0.014 m s^− 1^ based on previously published vertical swimming velocities and dominant krill body lengths in penguin diets^90,91^. Here, we assumed that krill only swam in the vertical and were passive drifters in the horizontal. While this vertical movement model could also be used for other zooplankton in the region, we focused on Antarctic krill due to their ecology role in the Southern Ocean, and particularly around the Peninsula^5,6^.

Krill were released along the continental shelf at 10 m and migrated down to 25, 50, 75, 100, and 150 m. Simulated krill were released every 8 km horizontally. Simulated microplastics were released at 10, 50, 100, and 150 m depth every 15, 20, 30, and 40 km in the horizontal, respectively, following the exponential decay of observed microplastics concentrations with depth^31^. Microplastics were modeled as passive particles and had no buoyancy or sinking rate. We also released simulated microplastics every 8, 10, 12, or 14 km horizontally within 50 km buffer zones of established research stations. Microplastics were released closer together (i.e. more particles were released) around stations and camps with higher peak populations following the methods of Gallagher et al.^77^. Research stations and camp locations were from the Council of Managers of National Antarctic Programs (COMNAP)^80^. Both simulated krill and microplastics included a vertical random walk to simulate vertical turbulence, which is parameterized within ROMS. Simulated krill and microplastics were advected at every model time step (50s) and positions were saved hourly.

Krill and microplastics were released weekly for 16 weeks starting on November 1 of each austral summer and were followed through March. We counted the number of simulated krill and microplastics on a 10 × 10 km (100 km^2^) grid. Krill and microplastics were not counted until they were advected for 5 days to avoid biasing counts based on release locations, following previous studies^49–51,77,83,89^.

### Model assumptions

Both of our simulated krill and microplastics models make several assumptions about their distributions and behavior. First, both krill and microplastics were released homogeneously throughout the study area in the horizontal, and at set depths within the upper 150 m in the vertical. Krill distributions and biomass are extremely heterogeneous along the Peninsula, especially between years of high krill recruitment, which only occur one to two times a decade^5,14,46,92–95^. Furthermore, if krill biomass is increasing or decreasing in the region is unclear^96,97^. Similarly, the true horizontal and vertical distributions and biomass of microplastics^25,34,39,98,99^ are poorly understood on broad spatiotemporal scales as most of the sampling comes from opportunistic samples, and are rarely systematic on the broad scales studied here. Therefore, we chose to release simulated krill and microplastics homogeneously in our simulations, rather than change the concentration of simulated particles released each simulated austral summer, to avoid biases in our results due to the high variability and uncertainties around krill and microplastic distributions, respectively. We accounted for this assumption in our analysis in two ways: (1) by not counting simulated krill or microplastics until they had been advected in the model for 5 days, and (2) using the hotspot persistence metric instead of the raw numbers of krill or microplastics counted within a grid cell.

The release depths of both simulated krill and microplastics are also relatively shallow in comparison to the bathymetry throughout the study area (> 2500 m off the shelf, upwards of 1500 m in the Bransfield Strait). We chose to limit our analysis to the upper 150 m to match known microplastic concentration data and limit our analysis to krill who are most likely to interact with these pollutants. These krill migration depths are also the most ecologically relevant to their predators such as *Pygoscelis* penguins^100^, and represent possible vectors for microplastic pollutants to higher trophic levels. Future studies should account for both the horizontal and vertical climatological distributions of krill and microplastics.

While krill are known to swim at speeds an order of magnitude greater than those modeled here^45,45,90^, and thus may be able to swim against slower currents temporarily, large-scale krill movement is poorly understood, especially at the spatial scales of ROMS, and a Brownian movement model would have resulted in no net movement on these scales. Therefore, we followed similar previous studies and assumed that krill horizontal movement was driven by advection^101–107^. We also did not account for krill ontogeny. While the larval distribution of krill eggs and larvae are more likely to be driven by horizontal advection, they also have different vertical movement behaviors than modeled here. Due to both ontogeny and a variety of other factors, krill DVM is highly variable on a variety of spatiotemporal scales^45,46,49,61,90,108^, while we assumed that this is a constant behavior. Future studies could add variability to this behavior. To account for variability in DVM behavior, we averaged the number of krill present within a 10 × 10 km grid cell over different DVM behaviors (see Analysis section below).

Similarly, our simulated microplastics were neutrally buoyant and did not have any sinking or floating behaviors, following previous studies^37,109^. Microplastic buoyancy and sinking behaviors are highly dependent on a myriad of factors including, but not limited to, the type, age, and shape of the particle^71^. Drifting sediment trap studies have illustrated that buoyant polymers are not more prominent in microplastics samples near the surface, suggesting that even buoyant microplastics can be found in the upper 150 m of the water column and are not limited to near-surface waters (upper 5 m)^31^. Therefore, our results may be representative of a range of polymer types and shapes. Allowing flexibility in both DVM behavior and particle characteristics will help improve future simulations.

### Analysis

We follow previously published methods^89,110^ to calculate simulated krill and microplastic hotspots. Krill and microplastic counts within grid cells were averaged within a single austral summer, transformed with a natural log, and limited to waters shallower than 2500 m. Within each 10 × 10 km grid cell, we determined how many counts (which occurred hourly during the austral summer, on the same timestamp the particle positions were saved) within a single year were above the average krill or microplastic counts plus one standard deviation. Hotspot persistence is defined as the fraction of these observations that are above this threshold (mean + 1 SD). We calculated hotspot persistence within years, and then across years by repeating the calculation across all eight simulated years, such that the interannual metric represented the fraction of years a grid cell had higher than average annual hotspot persistence (~ 25–30 days).

We ranked interannual hotspot persistence to assess relative risk of krill and microplastic potential interaction as function of their concentration, following previously published methods^41^. In brief, each 10 × 10 km grid cell was given a rank from 1 (lowest) to 5 (highest) separately for both krill and microplastic hotspots. Grid cells with zeros were given a rank of 1. Since hotspot persistence is calculated as a fraction, we chose to assign ranks based on quantiles such that grid cells with interannual hotspot persistence of < 0.25, 0.25–0.5, 0.5–0.75, and > 0.75 had ranks of 2, 3, 4, and 5, respectively. To quantify where the relative risk of krill and microplastic accumulation overlap, krill and microplastic hotspot ranks were averaged within grid cells.

We compared the resulting ranks across several groups to understand where krill and microplastics from various sources and depths have the highest likelihood of interacting. We calculated ranks for krill migrating within the surface ocean (upper 50 m), and to the deep ocean (> 50 m). We compared these krill hotspots to microplastic hotspots from simulated microplastics released in the (1) upper (< 50 m) and (2) deep (> 50 m) ocean; and (3) released near research stations and camps. We did not compare the ranks of krill migrating within the surface ocean (migrating to a maximum depth of 50 m) to microplastics released within the deep ocean (> 50 m) since we assumed that these krill and microplastics will not interact. We did compare deep migrating krill to microplastics released near the surface, since these deep migrating krill are vertically migrating near the surface at night to feed. The number of 10 × 10 grid cells with different ranks across comparisons were compared with a Kruskal-Wallis and pairwise Wilcoxon tests in R as appropriate.

### Validating krill hotspots

To validate the krill hotspots, we repeated this analysis with krill and chlorophyll-a hotspots. Krill are important phytoplankton grazers, especially in strong krill recruitment years, and are positively correlated with high chlorophyll-a concentrations within and across seasons^68,111,112^. We used the NOAA MSL12 multi-sensor DINEOF global 2 km gap-filled chlorophyll-a product from NOAA CoastWatch. This gap-filled product uses multiple satellites to reconstruct daily surface chlorophyll concentrations at a 2 km horizontal resolution^113^. We used the time period from 2018 to 2024 for this analysis. While this time frame only overlaps with some of the modeled years, we expect the long-term averages will represent the mean condition for validation purposes. Furthermore, satellite data can be inconsistent along the Peninsula during the austral summer, so we decided to use a gap-filled product, which was only available from 2018 onwards.

We averaged surface chlorophyll concentrations within our 10 × 10 km grid, and calculated hotspot persistence within (Figure S11) and across years (Figure S12). We ranked each grid cell and averaged the ranks across all krill and chlorophyll concentrations to compare them as described above (Figure S13-14). Kruskal-Wallis tests indicated that the ranks of the krill and chlorophyll hotspots did not differ significantly (chi-squared = 2.6, *p* = 0.11). Therefore, we can be reasonably confident that our krill hotspots are representative of areas where krill are likely to aggregate.

## Electronic Supplementary Material

Below is the link to the electronic supplementary material.

Supplementary Material 1.

## Data Availability

Simulated krill trajectories are available on the United States Antarctic Program Data Center (USAP-DC) at https://www.usap-dc.org/view/project/p0010349. Simulated microplastic trajectories are available upon request to the corresponding author. Chlorophyll data are available through via NOAA CoastWatch.
