# Supplementary material for: Interactions between vertical migration and local oceanography drive microplastic exposure for Antarctic krill
Source: Sci Rep. 2026 May 16;16:22217. doi: 10.1038/s41598-026-50531-0 (PMC13370005; doi:10.1038/s41598-026-50531-0)
Supplement: Supplementary file 1 — Supplementary Material 1. [file 41598_2026_50531_MOESM1_ESM.docx]

**Supplementary Material for**

Interactions between Vertical Migration and Local Oceanography Drive Microplastic Exposure for Antarctic Krill

Authors: Katherine L. Gallagher^1,2*^, Clara Manno^3^

Author Affiliations:

1. School of Marine and Atmospheric Sciences, Stony Brook University, Stony Brook NY 11794 USA
2. Current Address: IBSS Corporation in Support of NOAA Fisheries, Silver Spring, MD 20910
3. British Antarctic Survey, Cambridge, UK

*Corresponding Author: Katherine L Gallagher, [Katherine.L.Hudson@gmail.com](mailto:Katherine.L.Hudson@gmail.com)

Contents:

Supplementary Figures 1-13


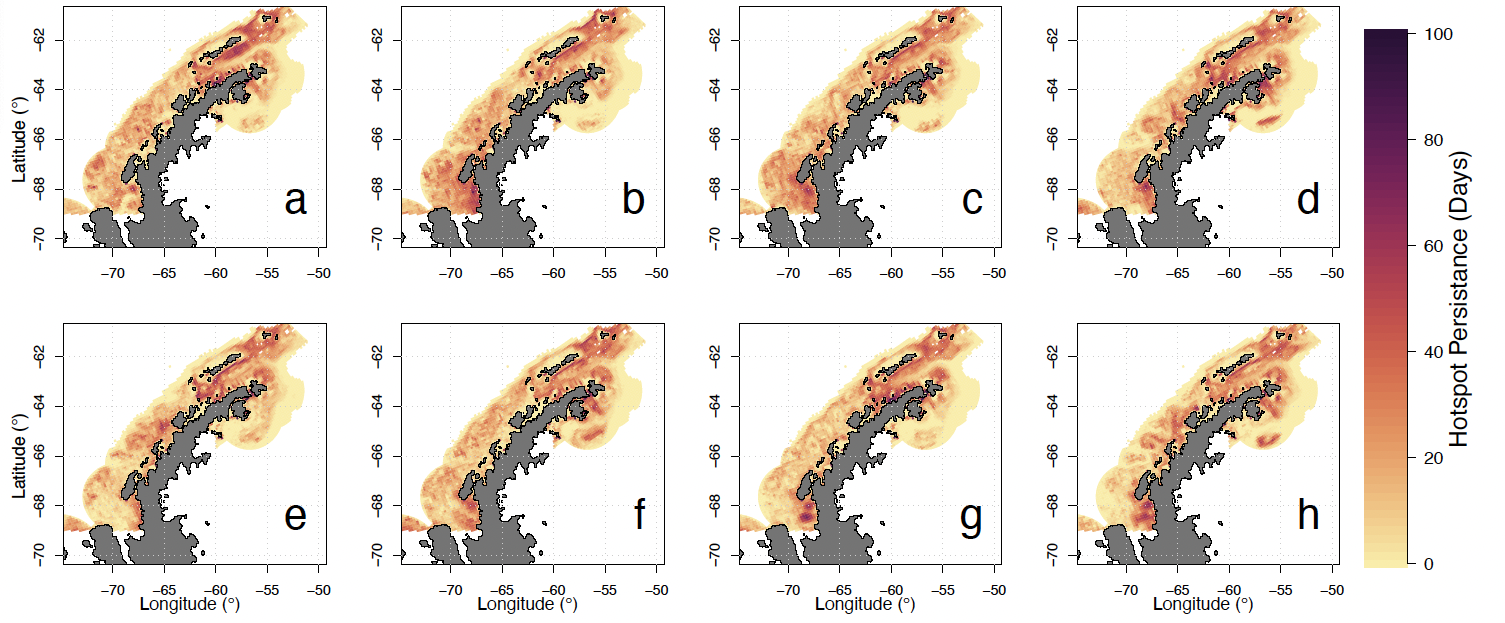


Figure S1. Krill hotspot persistence for krill migrating within the surface ocean (< 50 m) for 2006 - 2011 (a-f) and 2018 - 2019 (g-h) austral summers.


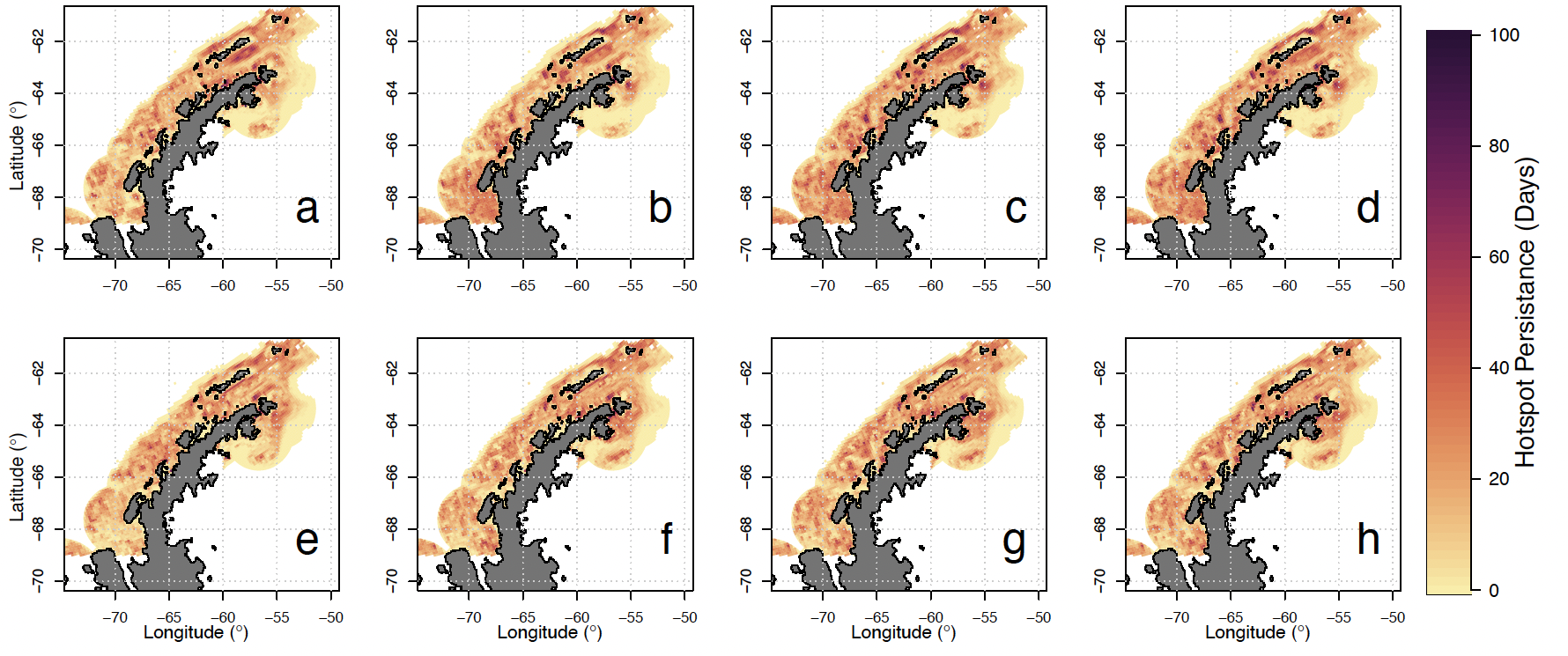


Figure S2. As in Figure S1 but for krill migrating to the deep ocean (> 50 m).


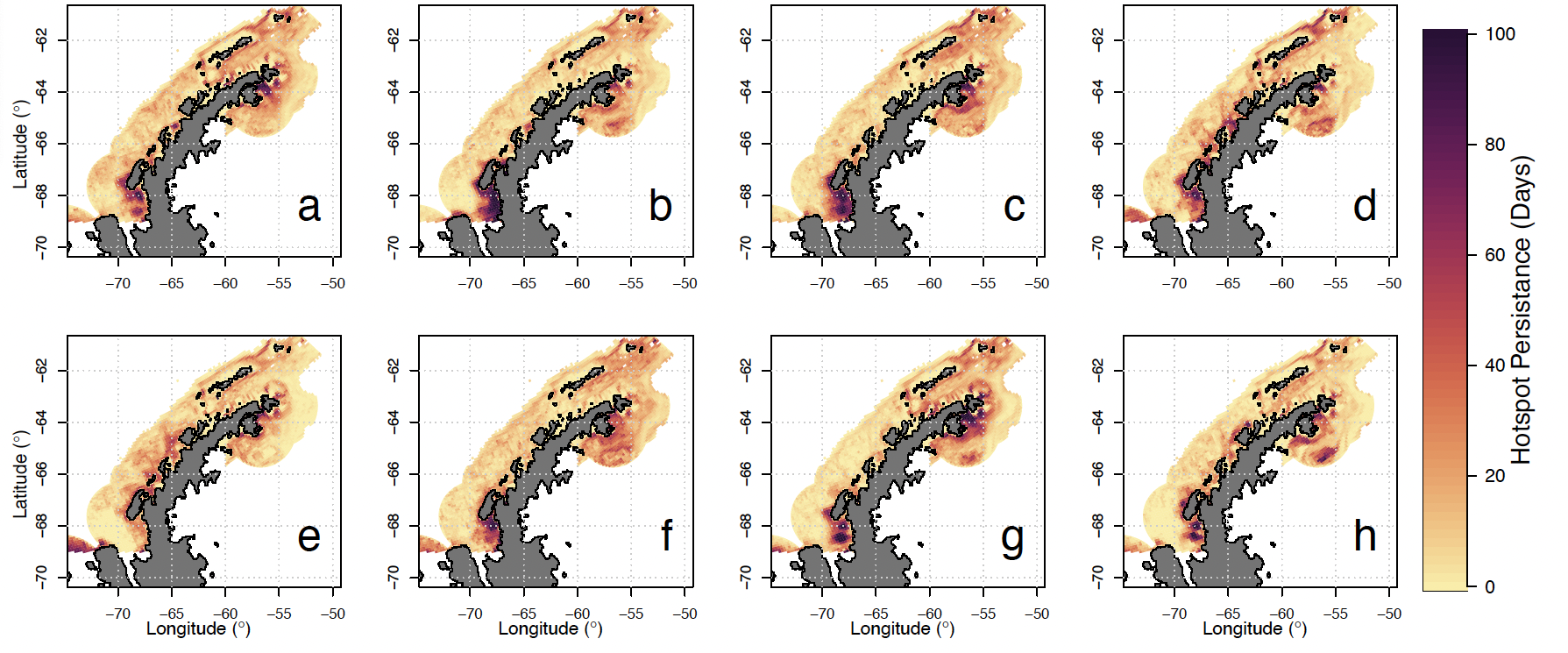


Figure S3. As in Figure S1 but for microplastics released within the surface ocean (< 50 m).


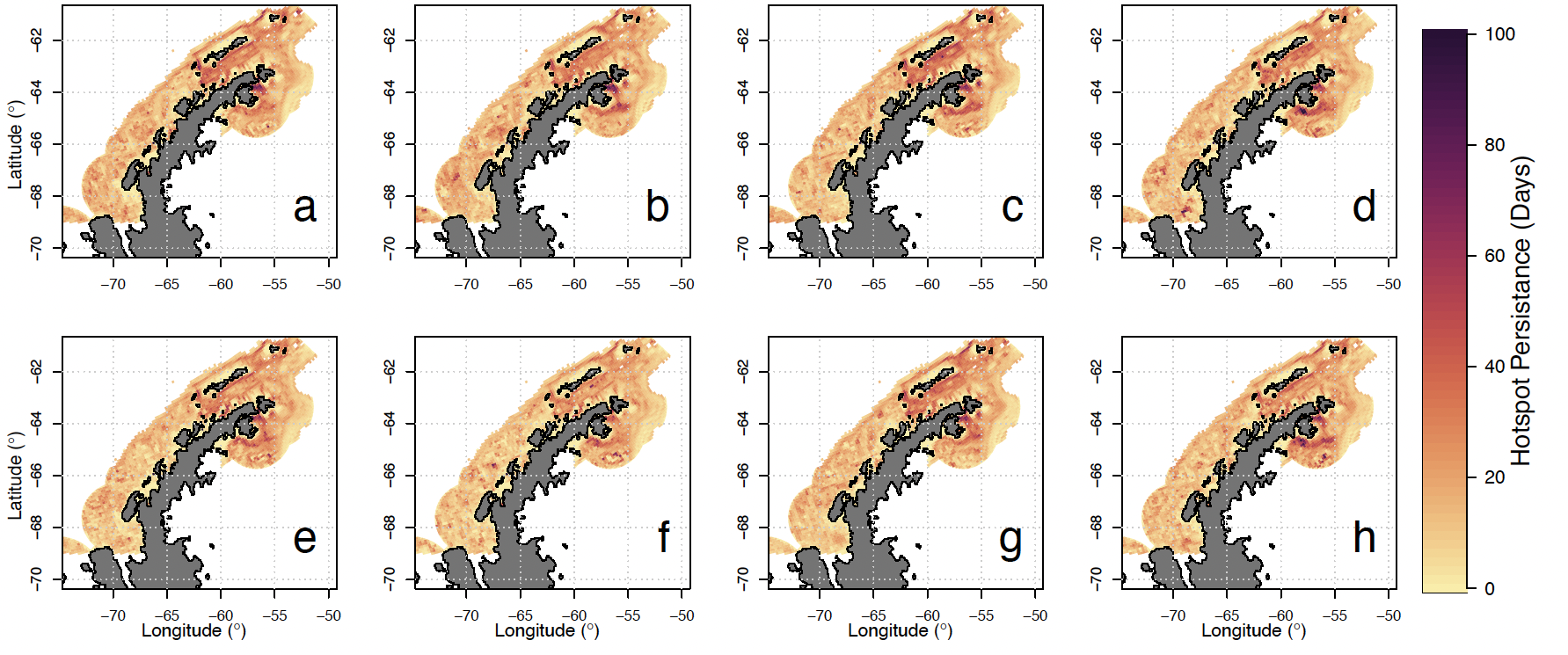


Figure S4. As in Figure S1, but for microplastics released in the deep ocean (> 50 m)


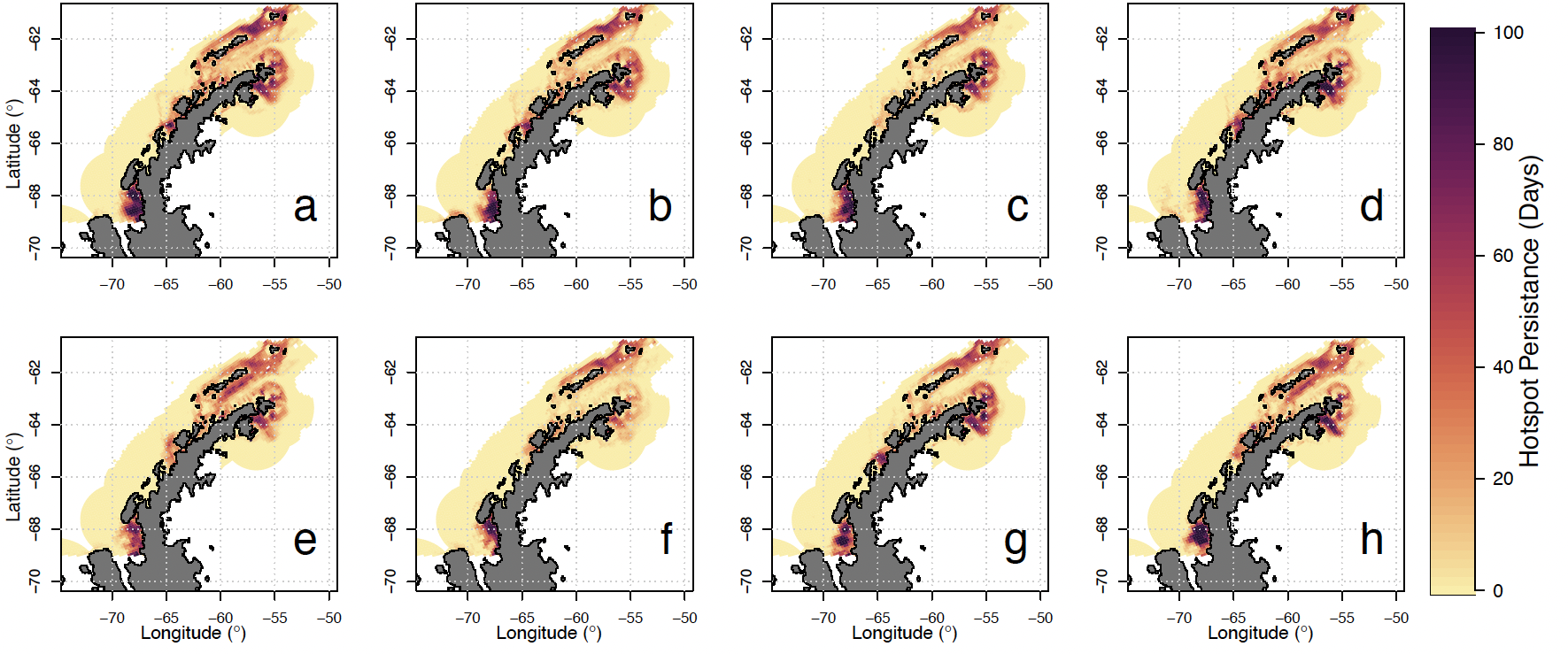


Figure S5. As in Figure S1, but for microplastics released near research stations and camps.


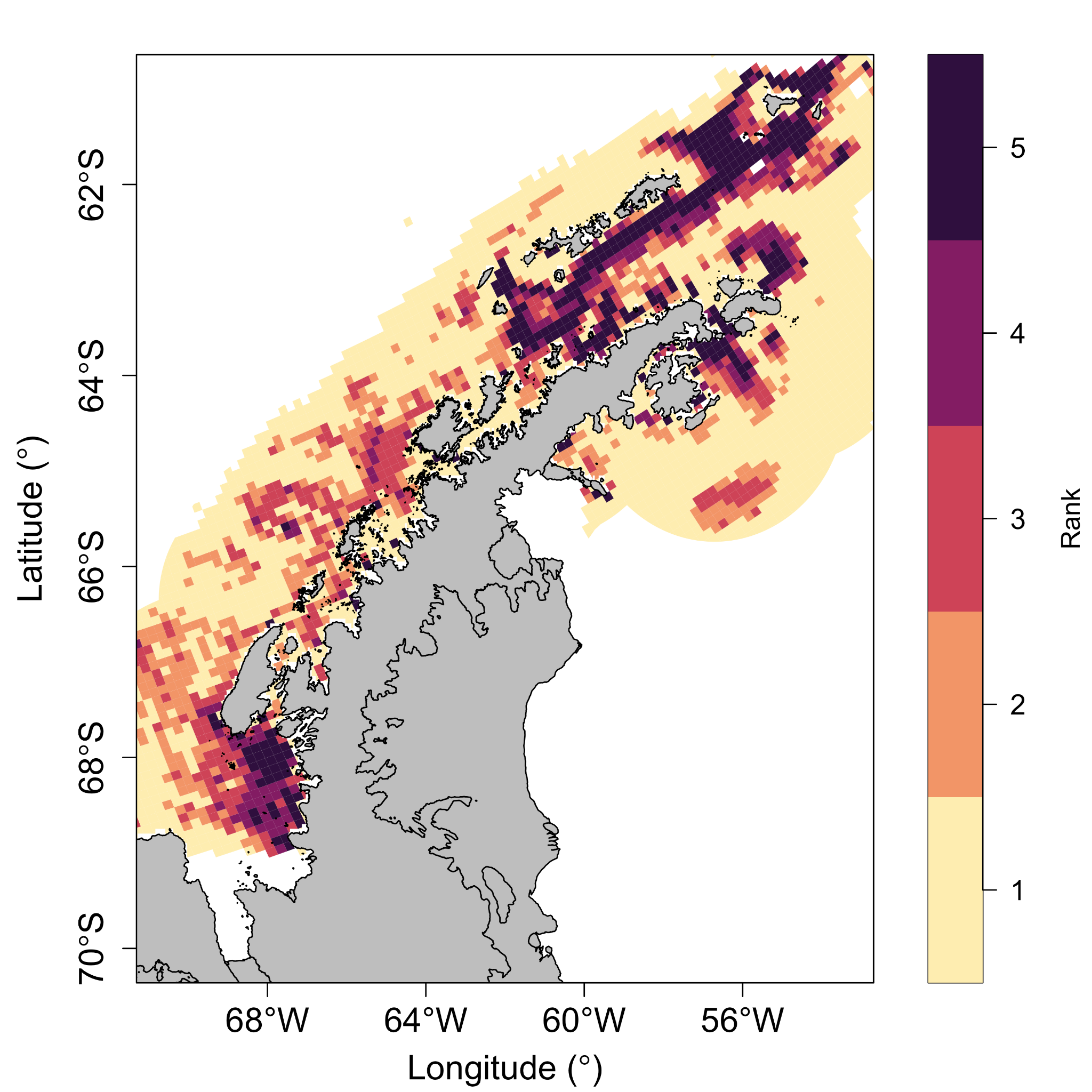


Figure S6. Mean rank of krill hotspots for krill migrating within the surface ocean (< 50 m).


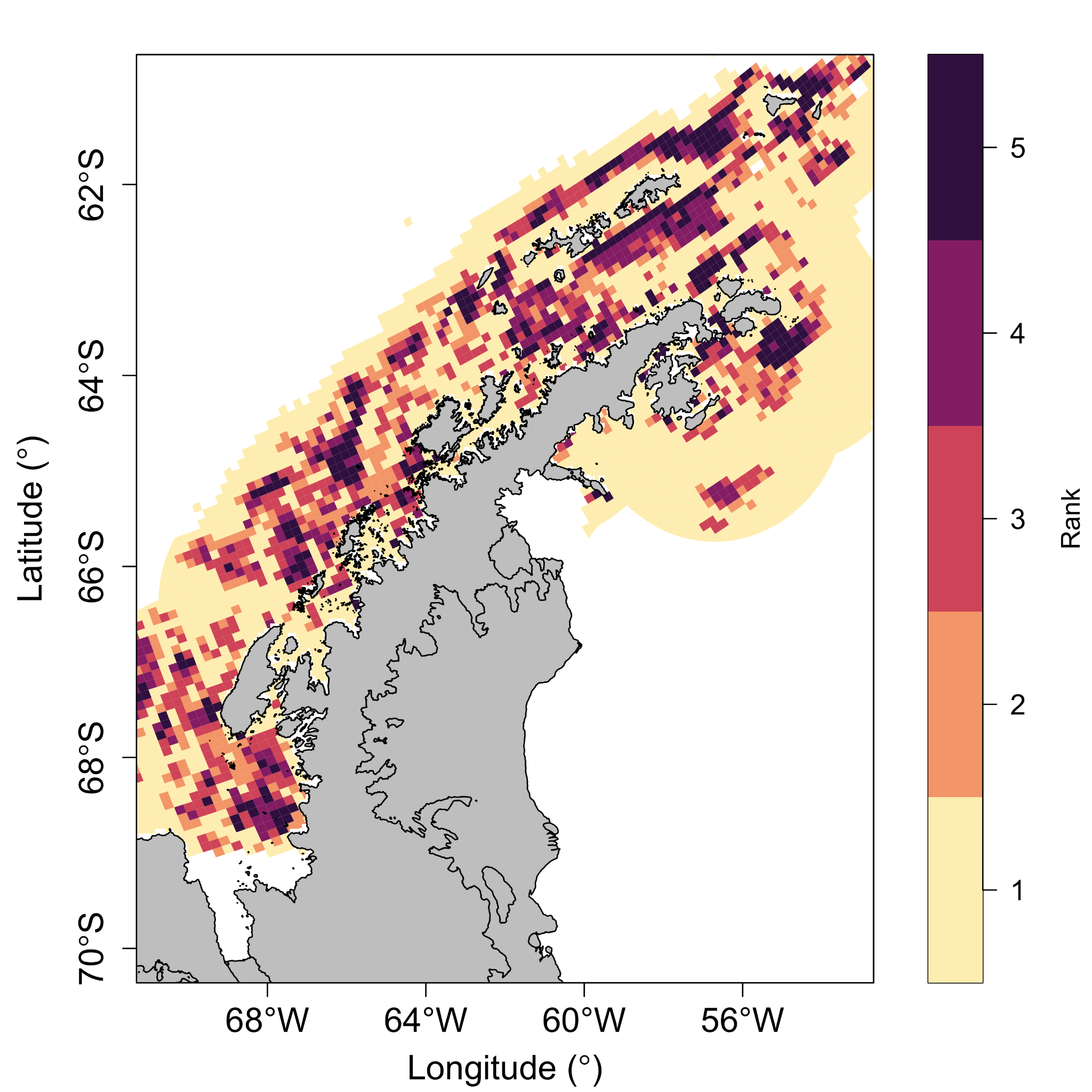


Figure S7. As in Figure S6, but for krill migrating into the deep ocean (> 50 m).


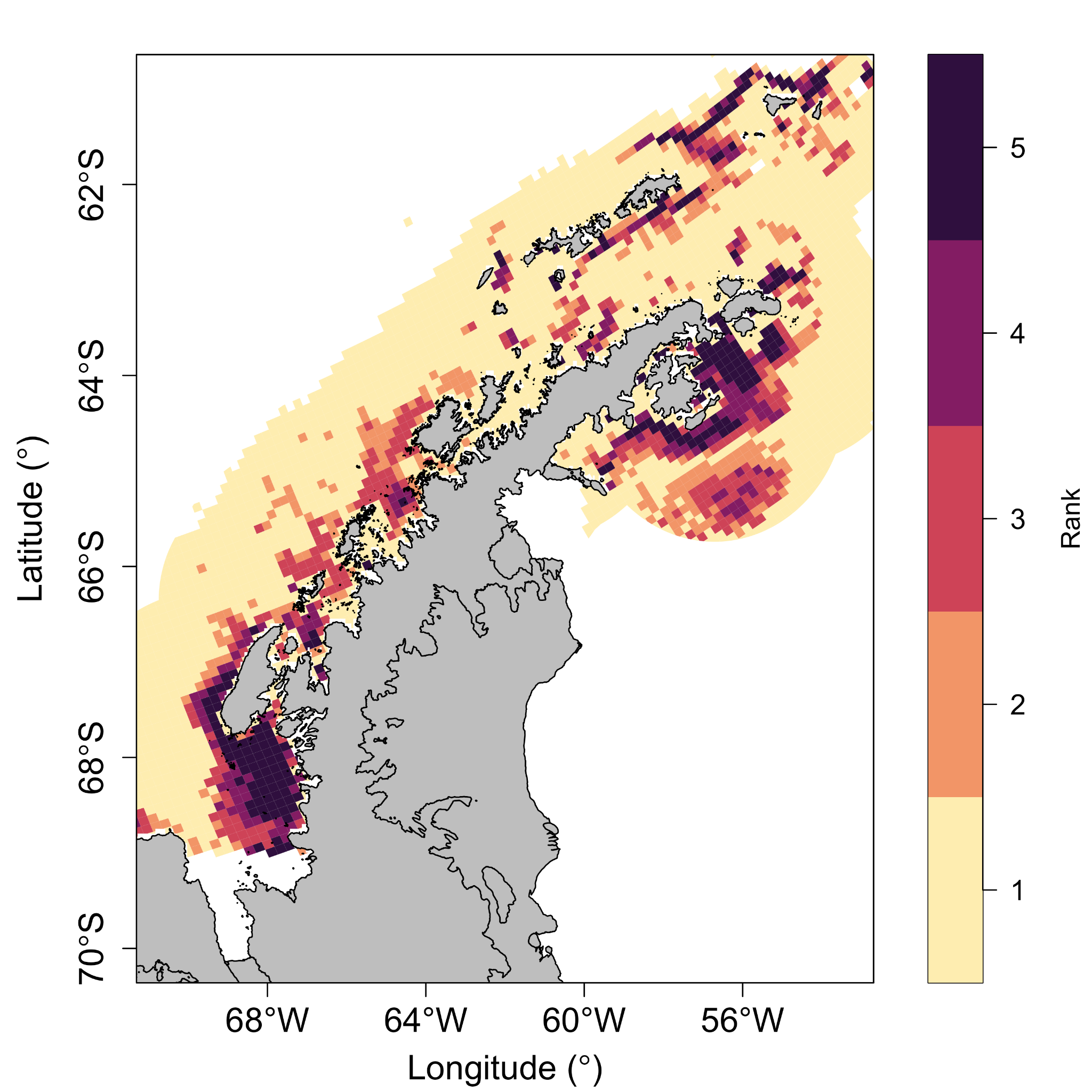


Figure S8. As in Figure S6, but for microplastics released in the surface ocean (< 50 m).


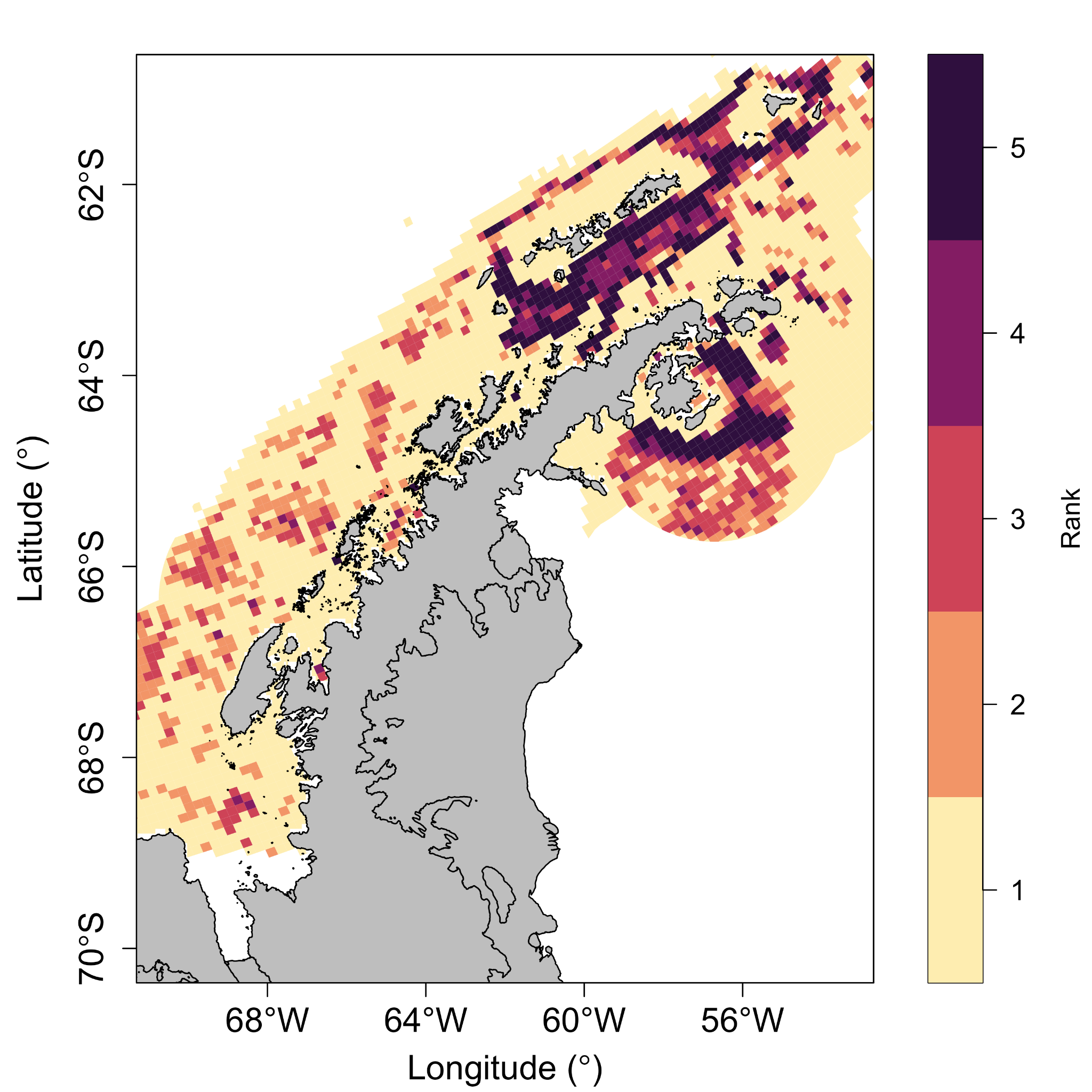


Figure S9. As in Figure S6, but for microplastics released in the deep ocean (> 50 m).


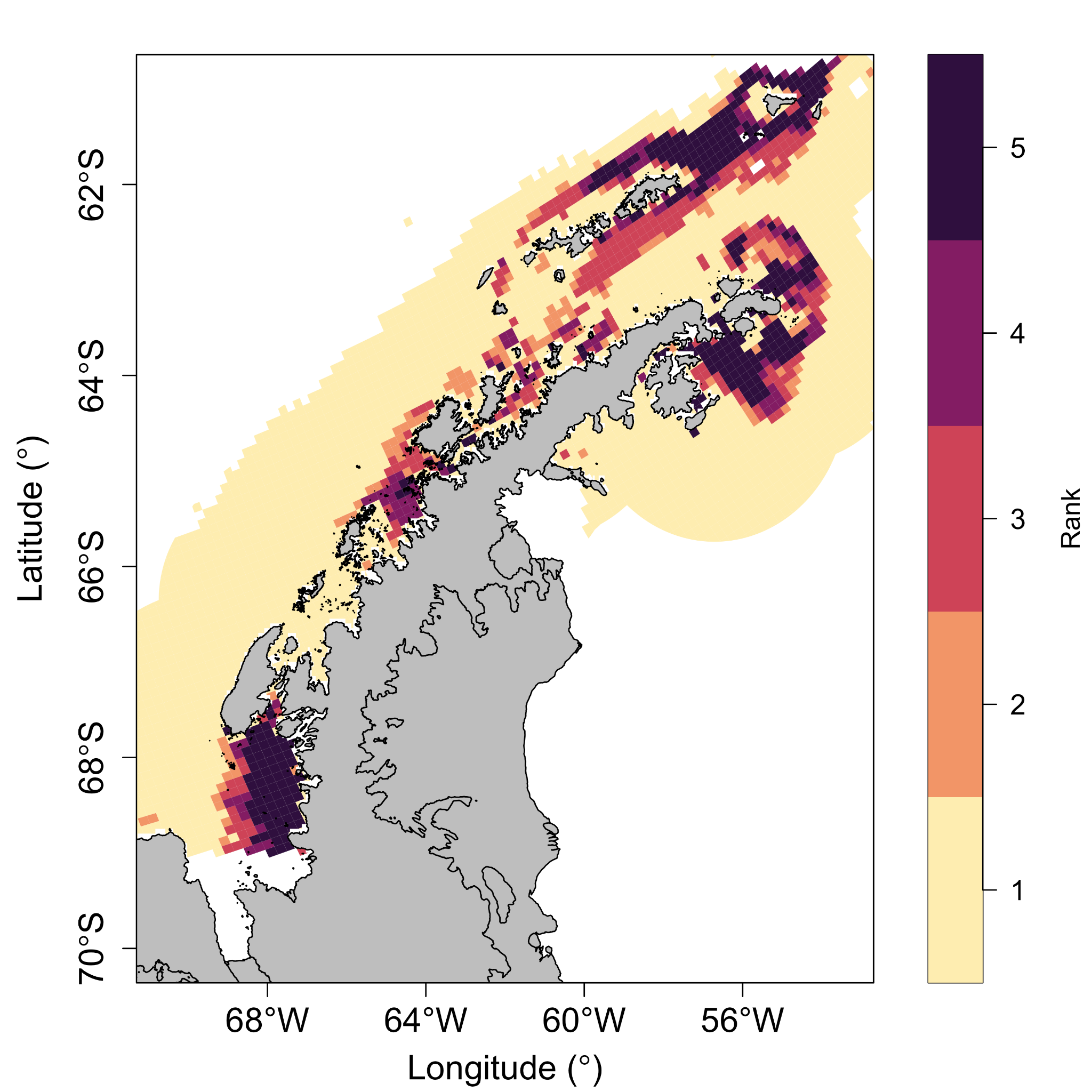


Figure S10. As in Figure 6, but for microplastics released around research stations and camps.


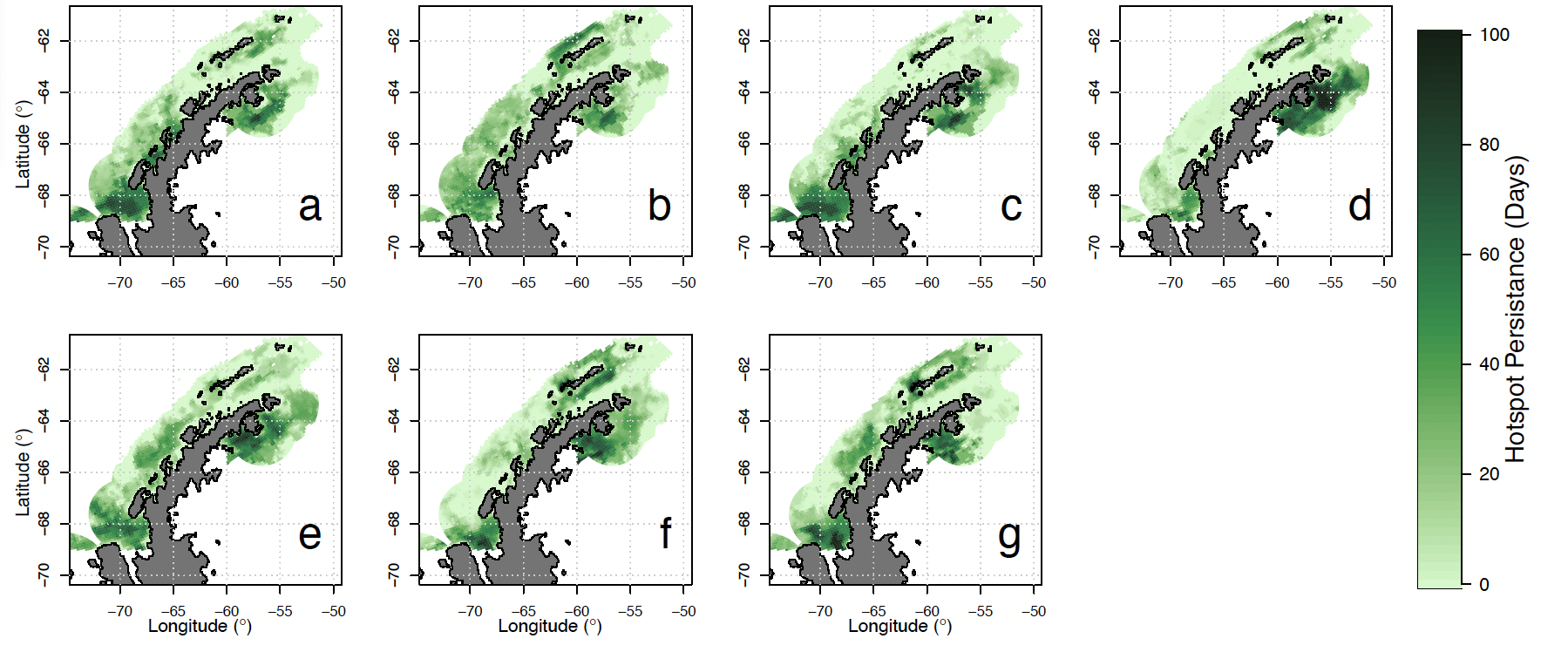


Figure S11. Hotspot persistence of surface chlorophyll for the austral summers of 2018 - 2024 (a-g).


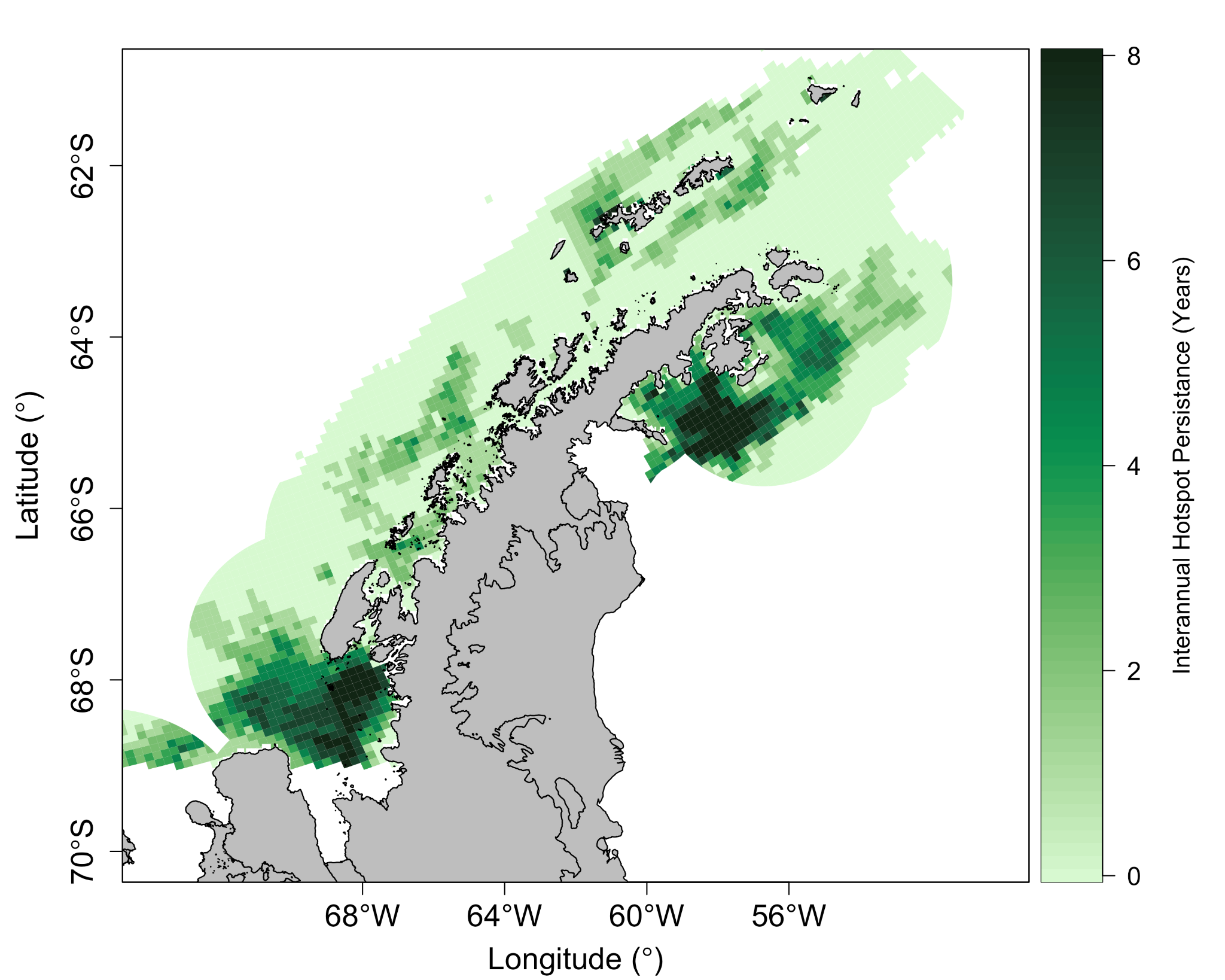


Figure S12. Surface chlorophyll interannual hotspot persistence across the study region.


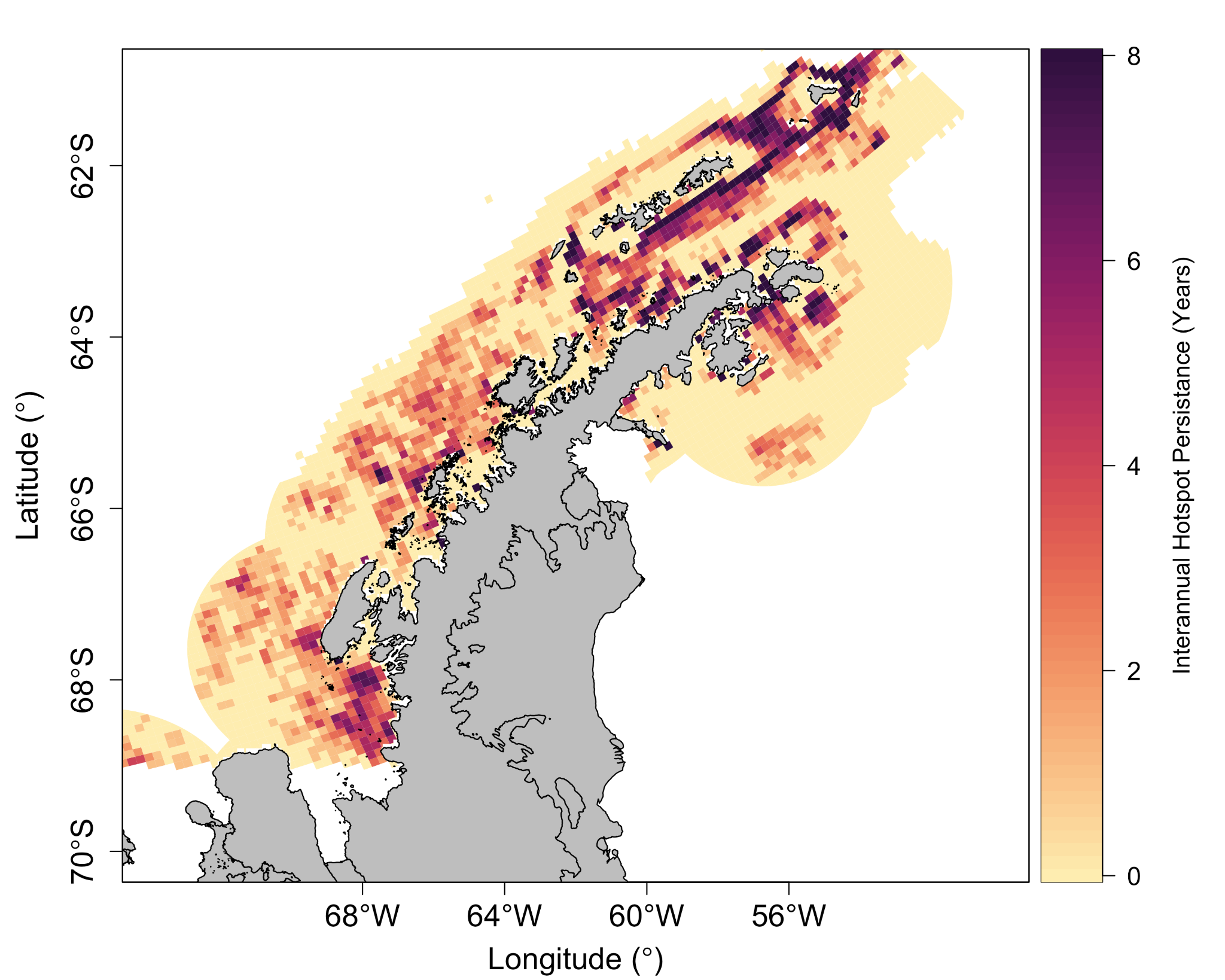


Figure S13. Interannual hotspot persistence in years for simulated krill across all migration depths.


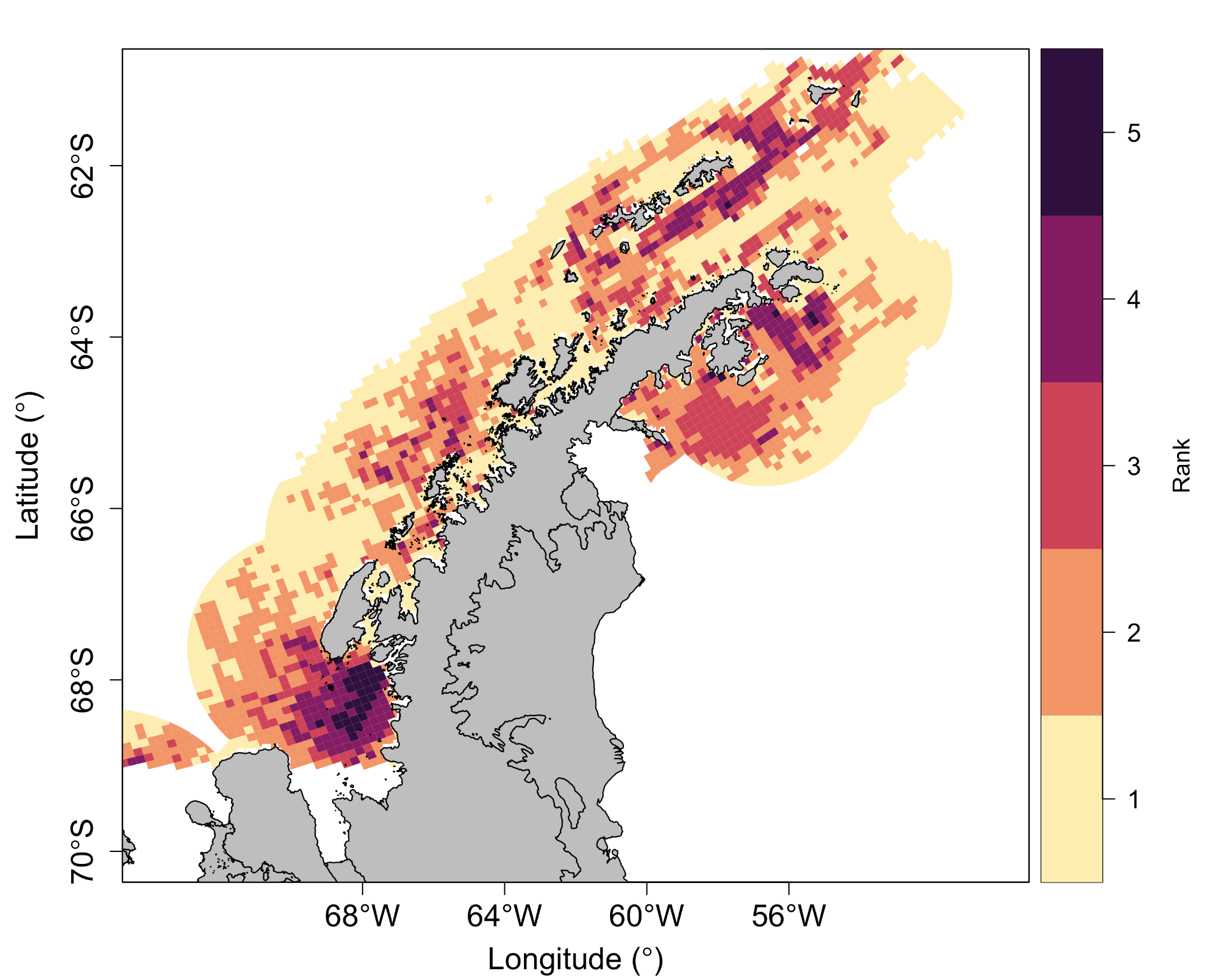


Figure S14. Overlap, determined by mean rank, of all krill and chlorophyll concentrations.
